# About the Cover Paul Gauguin (1848–1903). I Raro te Oviri (Under the Pandanus) (1891)

**DOI:** 10.3201/eid1004.AC1004

**Published:** 2004-04

**Authors:** Polyxeni Potter

**Affiliations:** * Centers for Disease Control and Prevention, Atlanta, Georgia, USA

**Figure:**
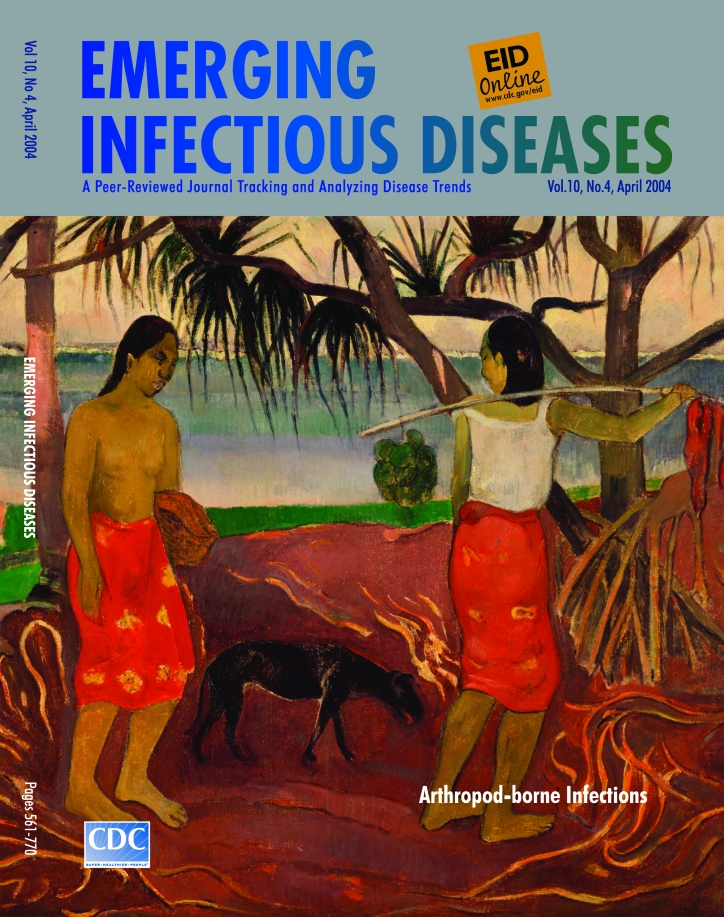
Paul Gauguin (1848–1903). I Raro te Oviri (Under the Pandanus) (1891) Oil on canvas (73.03 cm x 91.44 cm). Dallas Museum of Art, Foundation for the Arts Collection, gift of the Adele R. Levy Fund, Inc.

"Between me and the sky there was nothing except the high frail roof of the pandanus leaves, where the lizards have their nests," wrote Paul Gauguin in the autobiographical account of his first visit to Tahiti (*1*). Under the Pandanus (on this month's cover of Emerging Infectious Diseases) was painted shortly after Gauguin arrived on the islands in search of his famed reprieve from Western civilization.

Like many of his contemporaries, Gauguin became disillusioned with industrialized society whose intense focus on material gain seemed to strip life of its spiritual essence. Crushed under the yoke of familial responsibility, bewildered by the prosaic rules of art dealing, and stifled by societal constraints, Gauguin imagined a life uncluttered by the tedium of survival. He longed for a different world, one with just enough depth to sustain his most basic needs. Flat and two dimensional, this world would be filled with vibrant color and would celebrate the human spirit long lost under oppressive layers of cultural complexity and control.

While many rebel against civilization and espouse notions of an unspoiled haven, Gauguin set out to embody them in his life and work. A prosperous stockbroker and avid art collector, whose inventory included works by Daumier, Monet, Renoir, Manet, Cézanne, and Pissarro, he abandoned the Paris business scene and his brood of five children to devote his life to art at age 35 (*2*). Giving up comfort, commercial success, and artistic acclaim, he embraced isolation to know primitive idyll and find the core truth missing from his life.

He sought solace at first in Brittany among the peasants of the French countryside and then in the far away islands of the South Pacific, whose promise of paradise on earth lured many others, among them, Herman Melville, Mark Twain, and Robert Louis Stevenson. Under the blazing sun of the Polynesian islands, where as he put it in his copious writings, "…the material necessities of life can be had without money" (*3*), Gauguin articulated his artistic sentiment into original work that influenced generations to come. Synthesizing elements of his admired contemporaries, Cézanne, van Gogh, and others, and inspired by Japanese prints, folk art, and medieval stained glass, he created exuberant tableaux charged with sensuality and primal tension.

Gauguin's life as adoptive "savage" was one of unrelenting hardship, for the primitive idyll existed only in his inflamed imagination. From the moment he arrived on the islands, he was plagued by two of the many motivators of civilization, poverty and disease. Unable to afford even painting supplies and weakened by malnutrition and syphilis, he moved from Martinique to Tahiti and finally the Marquesas Islands, where he died at age 53.

Gauguin's art expressed his vision of the world. The edge of the canvas did not frame the images but rather opened them to wider exploration. Unspoiled nature was bountiful and generous, warm, forgiving, and open. Like his paintings, it had no boundaries, and its essence existed only in the imagination. Even as his body failed and his resources expired, its lure did not fray, nor did his zeal for it diminish.

Unlike civilized society, whose joyless monotone had alienated him, the primitive idyll had not been meddled with or manipulated. Unregulated and unrestrained, it followed nature's rhythm. Painted in startling, unnatural colors that punctuated the spiritual as well as the physical, it had a languid but steady beat. In the moist heat, laboriously outlined flat figures of humans and animals shared a communal living, even if it was not, as Gauguin wished it, altogether loving and harmless.

The prickly pandanus (screw pine), whose symbolic abundance pervades the painting on this issue's cover, is native to many Pacific archipelagoes, providing roof, sustenance, adornment, and medicine to generations of islanders (*4*). Filtering the sea breezes and moderating the tropical heat, the pandanus shelters the underbrush, which contains the complex ecosystem at the heart of the tropics' languid beat.

Undergrowth vegetation in tropical and subtropical areas is home to countless creatures (mammals, reptiles, birds) that sustain sandflies, ticks, fleas, and mosquitoes, whose complex natural cycles flourish in the heat and humidity so central to Gauguin's Eden. Unnoticed and unpainted, these vectors nurture the dark underpinning of untamed nature, including arthropod-borne disease: in this issue of Emerging Infectious Diseases alone, sleeping sickness in Uganda; dengue in Cuba, French Guiana, Bangladesh, and Myanmar; cutaneous leishmaniasis in Colombia; malaria in Western Kenya; West Nile virus in Guadeloupe; murine virus in the Canary Islands.
